# Supplementary material for: Intraspecific variation of phragmocone chamber volumes throughout ontogeny in the modern nautilid Nautilus and the Jurassic ammonite Normannites
Source: PeerJ. 2015 Oct 6;3:e1306. doi: 10.7717/peerj.1306 (PMC4614987; doi:10.7717/peerj.1306)
Supplement: Table S1 — Actual shell volumes were calculated based on measurement of weight of specimens with possible minimum shell density (2.54 g/cm3; Hoffmann & Zachow, 2011) and maximum shell density (2.62 g/cm3; Reyment, 1958) of Nautilus. [file peerj-03-1306-s001.docx]

| *Nautilus pompilius* | | | | | |
| --- | --- | --- | --- | --- | --- |
| Specimen | Measured weight of conch (g) | Estimated shell density (2.54 g/cm^3^) | Estimated shell density (2.62 g/cm^3^) | Measured volume from CT data (ml) | Estimated error (%) |
|  |  | Calculated volume (ml) | Calculated volume (ml) |  |  |
| 7 | 257.5 | 101.4 | 98.3 | 163.1 | 60.8-65.9 |
| 8 | 201.9 | 79.5 | 77.1 | 132.8 | 67.1-72.3 |
| 10 | 214.9 | 84.6 | 82.0 | 139.2 | 64.6-69.7 |
| 11 | 185.2 | 72.9 | 70.7 | 126.3 | 73.2-78.6 |
| 12 | 204.4 | 80.5 | 78.0 | 132.5 | 64.6-69.8 |
| 15 | 244.0 | 96.1 | 93.1 | 168.9 | 75.8-81.4 |
| 16 | 242.1 | 95.3 | 92.4 | 165.3 | 73.4-78.8 |
| 17 | 241.3 | 95.0 | 92.1 | 158.0 | 66.4-71.6 |
| 20 | 57.0 | 22.5 | 21.8 | 40.6 | 81.0-86.7 |
| 23 | 66.0 | 26.0 | 25.2 | 46.1 | 77.6-83.1 |
| 30 | 135.7 | 53.4 | 51.8 | 90.7 | 69.9-75.2 |
| 31 | 104.9 | 41.3 | 40.0 | 75.0 | 81.5-87.2 |
| 32 | 106.6 | 42.0 | 40.7 | 76.1 | 81.4-87.1 |
| 33 | 101.3 | 39.9 | 38.7 | 72.6 | 81.9-87.7 |
| 34 | 129.4 | 50.9 | 49.4 | 87.6 | 72.0-77.4 |
| 35 | 82.2 | 32.4 | 31.4 | 59.5 | 83.8-89.6 |
| 36 | 159.3 | 62.7 | 60.8 | 107.9 | 72.0-77.5 |
| 38 | 186.0 | 73.2 | 71.0 | 124.6 | 70.2-75.6 |
| 39 | 179.4 | 70.6 | 68.5 | 122.9 | 74.1-79.5 |
| 40 | 210.0 | 82.7 | 80.2 | 133.8 | 61.8-66.9 |
| 41 | 225.8 | 88.9 | 86.2 | 147.2 | 65.6-70.8 |
| 42 | 208.8 | 82.2 | 79.7 | 152.5 | 85.4-91.3 |
| 43 | 168.3 | 66.2 | 64.2 | 112.5 | 69.3-75.2 |
| 44 | 187.4 | 73.8 | 71.5 | 121.7 | 65.0-70.2 |
| 46 | 183.3 | 72.2 | 70.0 | 118.2 | 63.7-68.9 |
| 48 | 185.0 | 72.8 | 70.6 | 125.1 | 71.7-77.1 |
| 51 | 225.9 | 88.9 | 86.2 | 152.8 | 71.8-77.2 |
| 53 | 219.1 | 86.3 | 83.6 | 158.5 | 83.7-89.5 |
| 54 | 180.4 | 71.0 | 68.9 | 127.6 | 79.7-85.4 |
| 56 | 211.4 | 83.2 | 80.7 | 145.5 | 74.8-80.3 |
